# The association of organophosphate flame retardants (OPFRs) exposure on omega-3 fatty acids metabolism: evidence derived from the United States general population

**DOI:** 10.1093/toxres/tfaf119

**Published:** 2025-08-17

**Authors:** Ting-Hsuan Hsu, Hsiu-Yung Pan, Kai-Fan Tsai, Chia-Te Kung, Wan-Ting Huang, Huey-Ling You, Shau-Hsuan Li, Chin-Chou Wang, Wen-Chin Lee, Fu-Jen Cheng

**Affiliations:** Department of Emergency Medicine, Kaohsiung Chang Gung Memorial Hospital, Chang Gung University College of Medicine, No. 123, Dapi Rd., Niaosong Township, Kaohsiung County 833, Taiwan; Department of Emergency Medicine, Kaohsiung Chang Gung Memorial Hospital, Chang Gung University College of Medicine, No. 123, Dapi Rd., Niaosong Township, Kaohsiung County 833, Taiwan; Division of Nephrology, Department of Internal Medicine, Kaohsiung Chang Gung Memorial Hospital and Chang Gung University College of Medicine, No. 123, Dapi Rd., Niaosong Township, Kaohsiung County 833, Taiwan; Department of Emergency Medicine, Jen-Ai Hospital, No. 483, Dongrong Rd., Dali Dist., Taichung City 412224, Taiwan; Department of Emergency Medicine, Kaohsiung Chang Gung Memorial Hospital, No. 123, Dapi Rd., Niaosong Township, Kaohsiung County 833, Taiwan; Department of Laboratory Medicine, Kaohsiung Chang Gung Memorial Hospital and Chang Gung University College of Medicine, No. 123, Dapi Rd., Niaosong Township, Kaohsiung County 833, Taiwan; Department of Laboratory Medicine, Kaohsiung Chang Gung Memorial Hospital and Chang Gung University College of Medicine, No. 123, Dapi Rd., Niaosong Township, Kaohsiung County 833, Taiwan; Division of Hematology-Oncology, Department of Internal Medicine, Kaohsiung Chang Gung Memorial Hospital and Chang Gung University College of Medicine, No. 123, Dapi Rd., Niaosong Township, Kaohsiung County 833, Taiwan; Department of Occupational Medicine, Kaohsiung Chang Gung Memorial Hospital and Chang Gung University College of Medicine, No. 123, Dapi Rd., Niaosong Township, Kaohsiung County 833, Taiwan; Division of Nephrology, Department of Internal Medicine, Kaohsiung Chang Gung Memorial Hospital and Chang Gung University College of Medicine, No. 123, Dapi Rd., Niaosong Township, Kaohsiung County 833, Taiwan; Department of Emergency Medicine, Kaohsiung Chang Gung Memorial Hospital, Chang Gung University College of Medicine, No. 123, Dapi Rd., Niaosong Township, Kaohsiung County 833, Taiwan; Institute for Translational Research in Biomedicine, Kaohsiung Chang Gung Memorial Hospital, No. 123, Dapi Rd., Niaosong Township, Kaohsiung County 833, Taiwan

**Keywords:** organophosphate flame retardant exposure, omega-3 fatty acid, eicosapentaenoic acid, docosapentaenoic acid, diphenyl phosphate, bis(1,3-dichloro-2-propyl) phosphate

## Abstract

This study investigated the association between exposure to organophosphate flame retardants (OPFRs) and serum omega-3 fatty acid levels in the general U.S. population, using data from 1,350 adults in the 2011–2014 National Health and Nutrition Examination Survey (NHANES). OPFRs are widely used in consumer and industrial products, and emerging evidence has linked them to disruptions in lipid metabolism. In this study, urinary concentrations of five OPFR metabolites were analyzed in relation to serum levels of key omega-3 fatty acids, including eicosapentaenoic acid (EPA), docosahexaenoic acid (DHA), and docosapentaenoic acid (DPA), with adjustment for potential confounders. We observed significant negative associations between higher levels of diphenyl phosphate (DPhP) and the concentrations of EPA, DHA, and DPA. Similarly, bis(1,3-dichloro-2-propyl) phosphate (BDCPP) was negatively associated with EPA, bis(1-chloro-2-propyl) phosphate (BCEP) with DHA, and dibutyl phosphate (DBUP) with alpha-linolenic acid and DPA. Participants in the highest quartiles of DPhP and BDCPP exposure showed 18.2 and 18.4% lower EPA levels compared to the lowest quartiles, respectively. DHA levels declined by 17.5% with increasing DPhP and by 9.4% with sum of OPFRs (ΣOPFRs). These findings suggest that environmental OPFR exposure may interfere with omega-3 fatty acid metabolism and highlight potential metabolic and cardiovascular risks associated with these widely used flame retardants. These results underscore the importance of continued environmental monitoring and research into the health effects of OPFRs, particularly as their global use and human exposure continue to rise.

## Background

Organophosphate flame retardants (OPFRs) are new additives that help decrease the flammability of various consumer products and construction materials. Consequently, they are extensively utilized in furnishings, fabrics, building supplies, and the electronics industry.^1^ Owing to the persistence, bioaccumulation, and toxicity of polybrominated diphenyl ethers (PBDEs) – another type of flame retardant – in the environment, they have gradually been phased out of the flame-retardant market. Consequently, other cost-effective and efficient flame retardants have replaced them, with OPFRs being the most prominent. According to the 2024 European Chemicals Report, OPFRs currently account for approximately 30% of the global flame retardant market.^2^ With the increasing use of OPFRs, these compounds have been widely detected in various environmental media, including air, dust, water, soil, and sediments, as well as in human and non-human biota.^3^ In the United States, over 95% of the general population has measurable levels of OPFRs in urine, with bis(1,3-dichloro-2-propyl) phosphate (BDCPP, metabolite of TDCPP) and diphenyl phosphate (DPhP, metabolite of TPHP) being the most frequently detected metabolites, found in more than 90% of samples.^4^ In Australia, BDCPP and 1-hydroxy-2-propyl bis(1-chloro-2-propyl) phosphate (BCIPHIPP, metabolite of TCIPP) are the most commonly detected OPFR metabolites, each with detection frequencies exceeding 95%.^5^ OPFR metabolite levels tend to be higher in developed countries than in developing ones.^6^ As the usage of OPFRs rises, more research has been directed toward the potential health hazards posed by OPFRs. For instance, toxicological experiments have reported that exposure to OPFRs such as TDCPP and tris(2-chloropropyl) phosphate (TCPP) leads to increased secretion of estradiol (E2) and testosterone (T) in human adrenocortical carcinoma cell lines (H295R), and abnormal secretion of E2 and T in zebrafish.^7^ Additionally, exposure to tris(2-butoxyethyl) phosphate (TBEP) causes reproductive dysfunction and seminiferous tubule abnormalities in rats.^8^

Recent studies have increasingly focused on the impact of OPFR exposure on lipid and fatty acid metabolism. In vitro experiments by Hu et al. demonstrated that triphenyl phosphate (TPP) exposure in RAW264.7 macrophage cells induced endoplasmic reticulum (ER) stress and inflammatory responses, leading to the downregulation of lysophosphatidylcholine acyltransferase 3 (Lpcat3) and a subsequent decrease in fatty acid saturation.^9^ Similarly, exposure to tricresyl phosphate (TCP) has been shown to activate the pregnane X receptor (PXR), a nuclear receptor that regulates lipid metabolism, in human hepatocellular carcinoma HepG2 liver cells, resulting in elevated levels of free fatty acids and lipid droplet accumulation.^10^ Epidemiological studies further support these findings, with evidence linking OPFR exposure to elevated triglyceride levels and an increased risk of obesity.^11^ Our previous analysis of NHANES 2013–2014 data also revealed that urinary levels of DPhP, BDCPP, and bis(2-chloroethyl) phosphate (BCEP, metabolite of TCEP) were negatively associated with high-density lipoprotein cholesterol (HDL-C) levels.^12^

Polyunsaturated fatty acids (PUFAs) comprise two types: omega-6 and omega-3 fatty acids. They play several physiological roles, such as preserving cell membrane fluidity, reducing inflammatory responses, and lowering the release of proinflammatory cytokines.^13^ Omega-3 fatty acids are especially significant among these fatty acids. They encompass eicosapentaenoic acid (EPA) and docosahexaenoic acid (DHA), both of which possess important anti-inflammatory, antiplatelet, and antiarrhythmic effects, and are linked to lower mortality rates from cardiovascular diseases.^14^

While some research has suggested that exposure to specific endocrine-disrupting chemicals (EDCs) like phthalates may relate to circulating fatty acid levels, the connection between OPFR exposure and omega-3 fatty acid levels is still not well understood. This study aims to explore the relationship between OPFR exposure and omega-3 fatty acid levels in a representative sample of the U.S. population using data from the NHANES 2011–2014 cohort. By analyzing urinary OPFR metabolite levels and their correlation with serum omega-3 fatty acids, this study seeks to provide insights into the potential metabolic disruptions caused by OPFRs.

## Methods

### Study population

**Fig. 1:**
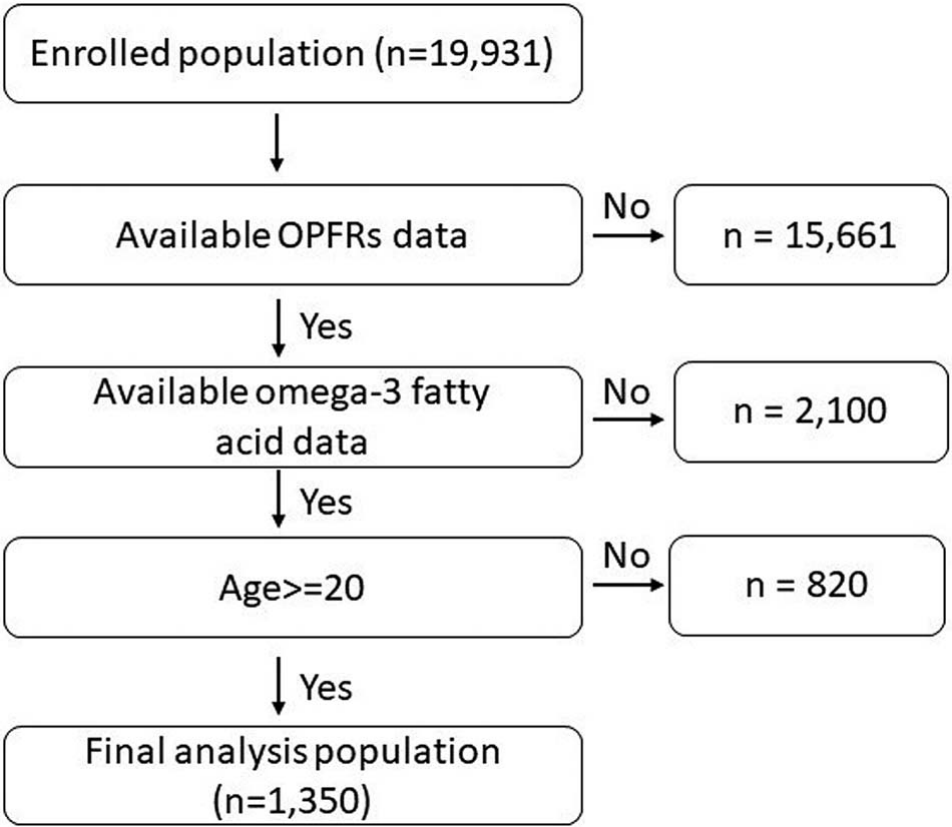
Participant flow chart algorithm.

The study population was identified from the 2011–2014 NHANES dataset, which is a nationwide survey recruiting a representative sample of the US household population to assess health and nutritional status. The 2011–2014 NHANES was approved by the Research Ethics Review Board of the US National Center for Health Statistics (Continuation of Protocol #2011–2017), and informed consent was obtained from all participants. The dataset and detailed survey protocols are provided on the NHANES website.^11^ In the 2011–2014 NHANES, a random subset of participants aged ≥6 years were selected for urinary OPFRs profile measurements, while lipid profiles were evaluated for participants in the same age range who provided serum specimens. For the purposes of this analysis, only adult participants adults aged ≥20 years, with both urinary OPFR profiles and serum omega-3 fatty acid data available from the 2011–2014 NHANES, were included (n = 1,350; Fig. 1).

### Measurement of urinary OPFR profiles

The measurement procedure for urinary OPFR concentrations in the 2011–2014 NHANES is provided on the NHANES website and has been described in previous reports.^4^^,^^15^^,^^16^ In summary, a 400 μL urine sample was used for analysis. To extract the analytes, enzymatic hydrolysis of the urinary conjugates and subsequent solid-phase extraction (SPE) with 60 mg of Strata XAW polymeric sorbent (Phenomenex, Torrance, CA, USA) were conducted. Reverse-phase high-performance liquid chromatography (HPLC) (Agilent 1,290; Agilent Technologies, Santa Clara, CA, USA) separated the target analytes in the extracts, which were then quantified using an isotope dilution-electrospray ionization tandem mass spectrometer (AB Sciex 5,500 Qtrap mass spectrometer; Applied Biosystems, Foster City, CA, USA).

In the 2011–2014 NHANES, eight OPFR metabolites were measured in urine samples for exposure assessment: bis(1-chloro-2-propyl) phosphate (BCPP), bis(1,3-dichloro-2-propyl) phosphate (BDCPP), bis(2-chloroethyl) phosphate (BCEP), diphenyl phosphate (DPhP), di-*n*-butyl phosphate (DBUP), di-*p*-cresyl phosphate, di-*o*-cresyl phosphate, and dibenzyl phosphate. This study focused on investigating the relationship between lipid metabolism and five OPFR metabolites, BCPP, BDCPP, BCEP, DBUP, and DPhP, which revealed detection rates of over 50% in the NHANES dataset. For non-detected samples, a value of LOD/√2 was assigned for the statistical analysis.

### Measurement of serum fatty acids

The analysis protocol for serum fatty acid levels from the 2011–2014 NHANES is available on the NHANES website.^15^ In summary, fatty acids were hydrolyzed from triglycerides, phospholipids, and cholesteryl esters by treatment with mineral acids, bases, or heat. Total fatty acids were hexane-extracted from serum samples using internal standards containing isotopically labeled fatty acids, and the extracts were subsequently derivatized with pentafluorobenzyl bromide and triethylamine to form pentafluorobenzyl esters. The reaction mixture was then injected into a capillary gas chromatography column to resolve the individual fatty acids, and an electron-capture negative-ion mass spectrometer (Agilent 7890A or 7890B; Agilent 5975C or 5977A; Agilent Technologies, Wilmington, DE, USA) was used to detect the fatty acids within 34 min. A total of 35 fatty acids were measured using selected ion monitoring in the 2011–2014 NHANES dataset, and this study focused on five omega-3 fatty acids (i.e. EPA, DHA, alpha-linolenic acid (ALN), stearidonic acid (SDA), and docosapentaenoic acid (DPA)). The NHANES quality assurance and quality control protocols met the 1988 Clinical Laboratory Improvement Amendment mandate.

### Collection of baseline characteristics

Following the NHANES research protocols, well-trained interviewers collected sociodemographic profiles through household interviews using standardized questionnaires and a computer-assisted personal interview system. In NHANES mobile examination centers, health technicians recorded body measurement profiles using standardized procedures. Other details of the recording procedures are provided on the NHANES website.^15^ Previous studies have indicated that factors such as age, sex, and ethnicity may be associated with dietary habits and omega-3 fatty acid intake, while the intake of PUFAs may influence serum omega-3 fatty acid concentrations.^17^^,^^18^ Therefore, age, sex, ethnicity, household income, alcohol consumption, body mass index (BMI), and estimated PUFA intake were obtained from the NHANES dataset for all participants.

### Statistical analysis

Continuous variables were expressed as medians with interquartile ranges (25th and 75th percentiles). To identify baseline covariates associated with omega-3 fatty acids and perform subgroup analyses, we divided the study population based on age (20–50 vs. >50 years), sex, ethnicity, annual household income (<45,000 vs. ≥45,000 USD), alcohol consumption (<12 vs. ≥12 drinks/year), and BMI (<25, 25–30, ≥30 kg/m^2^). We assessed the distribution of serum omega-3 fatty acid concentrations using the Shapiro–Wilk test. All variables yielded p-values <0.001, indicating significant deviations from normality. Comparisons of omega-3 fatty acid levels between subgroups were performed using the Mann–Whitney U test for two groups or the Kruskal–Wallis H test for three or more groups. Baseline covariates that showed statistically significant differences in omega-3 fatty acid concentrations between subgroups were included as confounding variables in the subsequent multiple linear regression models. Multiple linear regression analyses for complex survey data were conducted to examine the associations between urinary OPFR metabolite concentrations and omega-3 fatty acid levels. These models adjusted for baseline covariates and incorporated sampling weights according to the National Center for Health Statistics Analytical Guidelines. Although the distributions of omega-3 fatty acids were right-skewed and not strictly normal, they were approximately normal, with the skewness primarily driven by a small number of participants with elevated values. Therefore, we used the original (untransformed) omega-3 fatty acid values as outcome variables to facilitate clearer communication of results. Histograms of each omega-3 fatty acid are presented in Supplementary Fig. 1. Specifically, the sampling weights used in this study were derived from the “Surplus specimen B 11–12 2-yr weights” for the 2011–2012 cycle and the “Surplus specimen C 13–14 2-yr weights” for the 2013–2014 cycle. Before merging the two datasets, these sample weights were harmonized and then divided by two to appropriately account for the combined four-year survey period. Owing to their non-normal distribution, urinary OPFR metabolite concentrations were log-transformed prior to linear regression analysis. To further minimize the influence of outliers on the regression models, each OPFR concentration was also categorized into quartiles and incorporated into a trend analysis to examine the associations between increasing OPFR exposure and serum omega-3 fatty acid levels. Statistical significance was set at *P* < 0.05. All analyses were conducted using Statistical Product and Service Solutions software (version 22.0; IBM, Armonk, NY, United States).

## Results

### Levels of omega-3 fatty acids in participants

Serum omega-3 fatty acids in the different subgroups are listed in Table 1. The median (25, 75 percentile) values of serum EPA, DHA, ALA, SDA and DPA among the participants were 50.2 (33.4, 78.1) μmol/L, 142.0 (109.0, 196.0) μmol/L, 77.3 (56.5, 107.0) μmol/L, 3.0 (1.8, 4.9) μmol/L, and 47.9 (38.2, 61.2) μmol/L, respectively. Women (52.6 vs 47.3, *P* = 0.006), older adults (>50 years, 57.1 vs 44.2, *P* < 0.001), higher annual household income (≧4,5000 U.S. dollar, 55.8 vs 47.2, *P* < 0.001), and lower PUFA intake in 24-h dietary recall (≦0.15, 52.3 vs 47.2, 32.6–71.8, *P* = 0.004) participants had higher levels of EPA. Women, older adult, higher household income, lower alcohol consumption, and lower PUFA intake in 24-h dietary recall participants had higher levels of DHA.

**Table 1:** The median (25 and 75 percentile) of each omega-3 fatty acid in different subgroups.

	**Eicosapentaenoic acid (μmol/L)**			**Docosahexaenoic acid (μmol/L)**			**Alpha-linolenic acid (μmol/L)**			**Stearidonic acid (μmol/L)**			**Docosapentaenoic acid (μmol/L)**		
	**No**	**median (25 and 75 percentile)**	**p**	**No**	**median (25 and 75 percentile)**	***P***	**No**	**median (25 and 75 percentile)**	***P***	**No**	**median (25 and 75 percentile)**	***P***	**No**	**median (25 and 75 percentile)**	***P***
**Overall**	1,350	50.2 (33.4, 78.1)		1,346	142.0 (109.0, 196.0)		1,349	77.3 (56.5, 107.0)		1,224	3.0 (1.8, 4.9)		1,318	47.9 (38.2, 61.2)	
**Sex**			0.006			<0.001			0.386			0.799			0.15
male	641	47.3 (32.8, 74.5)		637	133.0 (102.0, 178.0)		641	76.7 (55.7, 106.5)		582	3.0 (1.82, 4.9)		625	48.4 (38.9, 62.1)	
Female	709	52.6 (35.4, 81.5)		709	153.0 (118.0, 211.0)		708	77.7 (57.2, 107.8)		642	2.9 (1.8, 4.9)		693	47.7 (37.7, 60.6)	
**Age (years**)			<0.001			<0.001			0.352			<0.001			<0.001
20–50	695	44.2 (30.3, 71.1)		693	131.0 (100.0, 178.0)		694	76.1 (56.6, 102.3)		623	2.6 (1.7, 4.5)		678	45.6 (36.7, 5,707)	
>50 (reference)	655	57.1 (39.0, 87.3)		653	159.0 (120.0, 217.0)		655	79.9 (56.1, 110.0)		601	3.2 (2.1, 5.2)		640	51.7 (41.0, 64.6)	
**Body mass index (kg/m2)**			0.651			0.223			0.003			0.006			0.637
<25	386	51.3 (32.9, 81.8)		384	148.0 (109.3, 214.8)		386	72.7 (54.4, 94.3)		342	2.7 (1.6, 4.3)		380	47.9 (37.7, 61.6)	
25–30	456	51.2 (33.5, 75.8)		455	141.0 (106.0, 193.0)		455	80.0 (57.1, 112.0)		418	3.0 (1.9, 5.2)		444	48.9 (38.5, 62.4)	
≧30	497	49.6 (34.5, 78.5)		496	140.5 (111.3, 186.0)		497	80.1 (57.8, 111.0)		454	3.2 (2.0, 5.0)		484	47.7 (38.6, 60.3)	
**Alcohol consumption (drink/year)**			0.134			0.002			0.674			0.02			0.513
12	330	47.8 (31.9, 71.9)		330	150.0 (118.0, 209.0)		330	77.9 (57.3, 110.0)		294	2.8 (1.8, 4.1)		325	47.7 (37.2, 61.6)	
≧12	919	50.9 (33.8, 78.8)		915	139.0 (105.0, 190.0)		918	77.2 (56.4, 105.0)		840	3.0 (1.8, 5.2)		898	47.9 (38.5, 60.9)	
**Annual Household income (USD)**			<0.001			<0.001			0.198			0.867			0.73
<45,000	645	47.2 (32.6, 70.3)		643	132.0 (101.0, 179.0)		644	77.1 (55.5, 104.8)		574	3.0 (1.9, 4.8)		628	47.8 (38.2, 60.1)	
> = 45,000	652	55.8 (119.0, 211.0)		651	154.0 (119.0, 211.0)		652	77.6 (57.5, 109.0)		601	2.9 (1.8, 5.0)		639	48.4 (38.3, 61.6)	
**Ethnicity**			<0.001			<0.001			<0.001			<0.001			<0.001
Mexican-American	175	37.2 (25.8, 52.6)		175	124.0 (101.0, 159.0)		174	85.5 (67.9, 121.0)		157	2.7 (1.4, 4.5)		171	45.5 (35.4, 57.1)	
Other Hispanic	140	48.4 (31.3, 66.7)		140	147.5 (117.3, 192.3)		140	81.0 (59.9, 116.8)		126	3.2 (1.8, 4.6)		137	48.1 (38.2, 62.8)	
Non-Hispanic White	594	54.1 (36.9, 82.2)		593	135.0 (1.1.0, 189.5)		594	78.0 (58.0, 106.3)		557	3.3 (2.1, 5.3)		577	50.6 (40.8, 63.8)	
Non-Hispanic Black	247	47.5 (32.8, 69.2)		245	148.0 (116.0, 195.5)		247	60.9 (47.6, 83.4)		224	2.5 (1.6, 3.9)		241	44.9 (36.2, 55.2)	
Other Race - Including Multi-Racial	194	60.5 (37.8, 106.0)		193	185.0 (134.0, 268.0)		194	82.7 (59.7, 122.3)		160	2.6 (1.6, 4.6)		192	47.8 (37.3, 63.7)	
**PUFA intake (gm)**			0.004			0.045			0.777			0.853			0.013
≦0.15	755	52.3 (34.4, 81.7)		753	144.0 (109.0, 198.0)		755	77.2 (56.8, 105.0)		691	3.0 (1.9, 4.8)		735	49.2 (39.0, 62.5)	
>0.15	503	47.2 (32.6, 71.8)		501	137.0 (105.5, 185.5)		502	77.7 (56.2, 109.0)		451	3.0 (1.8, 5.0)		494	46.6 (37.1, 60.4)	

### Association between DPhP exposure and the levels of omega-3 fatty acids

The detection frequency of the five major OPFR metabolites were shown in supplementary table 1. The adjusted regression coefficients (S.E.) for the differences in EPA, DHA, ALN, SDA, and DPA relative to a one-unit increase in log-transformed DPhP are summarized in Table 2. A one-unit increase in log DPhP levels was negatively associated with EPA (regression coefficient = −7.915; S.E. = 3.069; *P* = 0.015), DHA (regression coefficient = −18.096; S.E. = 5.881; *P* = 0.004), SDA (regression coefficient = −0.677; S.E. = 0.306; *P* = 0.034), and DPA (regression coefficient = −4.743; S.E. = 1.322; *P* = 0.001) in all participants. Subgroup analysis revealed that the effects on EPA levels (regression coefficient = −13.570; S.E. = 3.622; *P* = 0.001), SDA (regression coefficient = −1.516; S.E. = 0.523; *P* = 0.007), and DPA (regression coefficient = −7.311; S.E. = 2.106; *P* = 0.002) were more prominent in men.

**Table 2:** Adjusted regression coefficients (S.E.) for differences in omega-3 fatty acid relative to a one-unit increase in log10-transformed diphenyl phosphate (DPhP), with results weighted for sampling strategy.

	**Eicosapentaenoic acid (μmol/L)**			**Docosahexaenoic acid (μmol/L)**			**Alpha-linolenic acid (μmol/L)**			**Stearidonic acid (μmol/L)**			**Docosapentaenoic acid (μmol/L)**		
	**Unweighted no./Population size**	**Regression coefficient (S.E.)**	***P***	**Unweighted no./Population size**	**Regression coefficient (S.E.)**	***P***	**Unweighted no./Population size**	**Regression coefficient (S.E.)**	***P***	**Unweighted no./Population size**	**Regression coefficient (S.E.)**	***P***	**Unweighted no./Population size**	**Regression coefficient (S.E.)**	***P***
Overall	1,210/ 53,466,400	−7.92 (3.07)	0.015	1,148/ 51,175,298	−18.1 (5.88)	0.004	1,338 (57068532)	−4.99 (2.66)	0.069	1,127/ 49,353,600	−0.68 (0.31)	0.034	1,229/ 53,859,774	−4.74 (1.32)	0.001
**Sex**															
Male	578(25098217)	−13.57(3.62)	0.001	554(24390323)	−17.65(7.08)	0.018	635(26652434)	−7.72(4.1)	0.069	541(23559140)	−1.52(0.52)	0.007	585/25284654	−7.31(2.11)	0.002
Female	632(28368184)	−2.47(4.69)	0.602	594(26784975)	−17.86(8.03)	0.036	703(30416098)	−3.64(3.76)	0.34	586(25794460)	0.07(0.29)	0.819	644(28575120)	−2(1.19)	0.104
**Age (years)**															
20–50	628(28793617)	−8.00(3.47)	0.028	585(27142107)	−15.86(7.66)	0.046	688(30622159)	−5.57(5.34)	0.305	557(25985209)	−0.92(0.62)	0.148	636(29055012)	−3.18(1.67)	0.066
>50	582(24672783)	−6.3(5.76)	0.282	563(24033191)	−19.82(7.31)	0.013	650(26446373)	−3.75(4.24)	0.384	570(23368390)	−0.43(0.31)	0.178	593(24804763)	−6.07(1.61)	0.001
**Body mass index (kg/m** ^**2**^**)**															
<25	344(14680564)	−9.57(4.48)	0.04	320(13835737)	−13.33(7.09)	0.069	386(15584032)	−10.16(6.68)	0.143	309(13025788)	−1.24(1.21)	0.313	354(14845303)	−2.31(2.39)	0.342
25–30	413(19028922)	−2.68(3.94)	0.502	393(18186051)	−16.82(9.04)	0.072	455(20343822)	−1.87(4.34)	0.669	391(17922549)	−0.44(0.38)	0.26	416(19124390)	−4.87(2.01)	0.021
≧30	446(19611553)	−14.16(5.11)	0.009	428(19008148)	−22.35(9.03)	0.019	497(21140679)	−3.49(6.38)	0.588	427(18405253)	−0.48(0.27)	0.079	452(19744719)	−7.29(2.34)	0.004
**Alcohol consumption (drink/year)**															
<12	289(10184079)	−6.54(6.34)	0.311	289(10184079)	−20.92(8.95)	0.026	327(11054304)	−3.12(5.27)	0.558	292(9998263)	−0.01(0.21)	0.649	307(10654708)	−3.31(2.47)	0.191
≧12	862(41115945)	−7.81(3.15)	0.019	859(40991219)	−16.28(6.32)	0.015	913(42559157)	−5.62(3.23)	0.091	835(39355337)	−0.86(0.4)	0.04	865(41072743)	−5.52(1.48)	0.001
Income (USD)															
<45,000	604(20765108)	−4.77(4.49)	0.296	575(19834326)	−14.92(6.55)	0.03	639(21772125)	−7.15(3.67)	0.06	534(18692240)	−0.72(0.35)	0.047	589(20351227)	−3.31(1.78)	0.071
> = 45,000	606(32701293)	−10.45(4.11)	0.016	573(31340972)	−19.38(7.29)	0.012	647(33725326)	−2.47(4.74)	0.605	547(29284015)	−0.6(0.59)	0.322	594(32097279)	−5.19(2.30)	0.031
**Ethnicity**															
Mexican-American	150(4404881)	−1.42(3.32)	0.676	139(4046923)	−21.92(8.9)	0.03	171(5006399)	−8.17(5.78)	0,181	139(4149747)	−0.54(0.47)	0.279	157(4702000)	−5.35(2.81)	0.08
Other Hispanic	110(2876210)	11.3(3.83)	0.011	104(2666953)	2.51(11.96)	0.837	139(3563313)	4.25(4.66)	0.376	115(2761828)	0.30(0.35)	0.395	126(3248913)	2.82(1.93)	0.164
Non-Hispanic White	562(38067694)	−8.04(3.8)	0.043	543(36842123)	−15.45(7.64)	0.052	591(39536591)	−5.31(4.0)	0.194	530(35419924)	−0.82(0.42)	0.062	553(37780032)	−5.37(1.85)	0.007
Non-Hispanic Black	222(4371286)	−6.66(4.75)	0.176	213(4162272)	−15.91(7.56)	0.048	245(4805034)	−1.66(3.9)	0.674	204(3918381)	−0.27(0.3)	0.366	222(4341893)	−3.58(1.87)	0.07
Other Race - Including Multi-Racial	166(3746329)	−27.04(10.17)	0.014	149(3457027)	−51.97(14.15)	0.001	192(4157195)	−8.19(8.18)	0.327	139(3103719)	−0.21(0.51)	0.681	171)378636)	−4.77(2.5)	0.069

### Association between BDCPP exposure and the levels of omega-3 fatty acids

The adjusted regression coefficients (S.E.) for changes in EPA, DHA, ALN, SDA, and DPA in relation to a one-unit increase in log-transformed BDCPP are shown in Table 3. A one-unit rise in log BDCPP levels was significantly associated with lower EPA levels (regression coefficient = −9.047; S.E. = 3.988; *P* = 0.03). Further subgroup analyses indicated stronger effects in men (regression coefficient = −8.803; S.E. = 3.951; *P* = 0.033), younger adults aged 20–50 years (regression coefficient = −11.304; S.E. = 4.575; *P* = 0.019), and individuals with lower household income (regression coefficient = −10.660; S.E. = 4.730; *P* = 0.031).

**Table 3:** Adjusted regression coefficients (S.E.) for differences in omega-3 fatty acid relative to a one-unit increase in log10-transformed bis(1,3-dichloro-2-propyl) phosphate (BDCPP), with results weighted for sampling strategy.

	**Eicosapentaenoic acid (μmol/L)**			**Docosahexaenoic acid (μmol/L)**			**Alpha-linolenic acid (μmol/L)**			**Stearidonic acid (μmol/L)**			**Docosapentaenoic acid (μmol/L**		
	**Unweighted no./Population size**	**Regression coefficient (S.E.)**	**p**	**Unweighted no./Population size**	**Regression coefficient (S.E.)**	***P***	**Unweighted no./Population size**	**Regression coefficient (S.E.)**	***P***	**Unweighted no./Population size**	**Regression coefficient (S.E.)**	***P***	**Unweighted no./Population size**	**Regression coefficient (S.E.)**	***P***
**Overall**	1,210/ 53,466,400	−9.05 (3.99)	0.03	1,148/ 51,175,298	−7.77 (5.06)	0.135	1,338 (57068532)	−3.84 (3.69)	0.306	1,127/ 49,353,600	−0.77 (0.48)	0.114	1,229/ 53,859,774	−2.63 (1.32)	0.055
**Sex**															
Male	578/25098217	−8.80(3.95)	0.033	554/24390323	−10.06(6.74)	0.145	635(26652434)	−8.28(6.63)	0.221	541(23559140)	−1.74(0.86)	0.053	585(25284654)	−3.17(1.93)	0.11
Female	632/28368184	−8.28 (5.63)	0.151	594/26784975	−5.3(8.2)	0.523	703(30416098)	−4.60(4.73)	0.923	586(25794460)	0.02(0.38)	0.966	644(28575120)	−1.80(1.38)	0.202
**Age (years)**															
20–50	628/28793617	−11.30 (4.58)	0.019	585/27142107	−7.01/7.27	0.342	688(30622159)	−6.16(6.95)	0.382	557(25985209)	−1.57(0.97)	0.114	636(29055012)	−2.63(1.73)	0.137
>50	582/24672783	−4.17 (5.78)	0.476	563/24033191	−5.86/8.35	0.488	650(26446373)	−1.94(3.42)	0.575	570(23368390)	−0.001(0.25)	0.998	593(24804762)	−2.35(1.93)	0.232
**Body mass index (kg/m** ^**2**^ **)**															
<25	344(14680564) -	−7.84 (5.95)	0.199	320/13835737	−3.25/11.16	0.773	386(15584032)	−5.07(8.67)	0.563	309(13025788)	−1.6(1.39)	0.259	354(14845303)	−0.94(2.21)	0.674
25–30	413(19028922)	−6.91 (4.71)	0.153	393/18186051	−5.33/5.88	0.372	455(20343822)	−10.18(4.27)	0.022	391(17922549)	−0.57(0.53)	0.285	416(19124390)	−4.85(2.15)	0.031
≧30	446 (19611552)	−14.13 (7.04)	0.053	428/19008148	−12.49/8.39	0.147	497(21140679)	3.20(5.99)	0.597	427(18405263)	−0.5(0.86)	0.568	452(19744719)	−2.87(1.93)	0.147
**Alcohol consumption (drink/year)**															
<12	289 (10184079)	−3.57(8.97)	0.693	289 (10184079)	−10.14/11.88	0.4	327 (11054304)	−2.51(5.42)	0.646	292(9998263)	−0.54(0.27)	0.049	307(10654708)	−2.28(1.92)	0.244
≧12	862 (41115944)	−8.92 (3.97)	0.032	859 (40991219)	−6.43(5.21)	0.226	913(42559157)	−3.35(4.48)	0.46	835(39355337)	−0.82(0.6)	0.178	865(41072743)	−2.75(1.48)	0.071
**Income (USD)**															
<45,000	604(20765107)	−10.66(4.73)	0.031	575/19834326	−9.97(5.66)	0.088	639(21772125)	−4.36(5.33)	0.419	534(18692240)	−0.83(0.74)	0.269	589(20351227)	−1.35(1.68)	0.43
> = 45,000	606 (32701293)	−8.23 (5.27)	0.128	573/31340972)	−4.51(7.87)	0.571	647(33725326)	−3.04(6.42)	0.639	547(29284015)	−0.77(0.69)	0.274	594(32097279)	−3.34(1.69)	0.056
**Ethnicity**															
Mexican-American	150(4404881)	1.75 (2.86)	0.726	139(4046923)	−18.43(5.51)	0.006	171(5006399)	−5.41(7.95)	0.508	139(4149747)	- 0.16(0.71)	0.822	157(4702000)	0.99(2.18)	0.657
Other Hispanic	110(2879209)	−9.93(6.78)	0.165	104(2666953)	−27.79(15.09)	0.087	139(3563313)	−6.31(7.16)	0.392	115(2761828)	−0.8(0.51)	0.139	126(3248913)	−3.33(3.21)	0.317
Non-Hispanic White	562(38067694)	−8.95(4.67)	0.065	543(36842123)	−2.33(6.15)	0.708	591(39536591)	−2.37(4.97)	0.637	530(35419924)	−0.87(0.63)	0.178	553(37780032)	−2.82(1.68)	0.103
Non-Hispanic Black	222(4371286)	−5.55 (5.74)	0.345	213(4162272)	−12.75(7.89)	0.121	245(4805034)	−6.70(4.29)	0.132	204(3918381)	−0.31(0.29)	0.294	222(4341893)	−3.17(2.19)	0.162
Other Race - Including Multi-Racial	166(3746329)	−23.77(10.39)	0.032	149(3457027)	−38.58(13.09)	0.007	192(4157195)	−13.52(5.77)	0.028	139(3103719)	−0.85(0.45)	0.074	171(3786936)	−4.03(2.28)	0.091

### Association between BCPP exposure and the levels of omega-3 fatty acids

The adjusted regression coefficients (S.E.) for changes in EPA, DHA, ALN, SDA, and DPA with a one-unit rise in log-transformed BCPP are presented in Table 4. A single unit increase in log BCPP levels was linked to a significant decrease in SDA (regression coefficient = −0.760; S.E. = 0.266; *P* = 0.007) and DPA (regression coefficient = −3.023; S.E. = 1.447; *P* = 0.045) across all participants. In subgroup analyses, younger adults (20–50 years) exhibited more pronounced effects on EPA (regression coefficient = −8.216; S.E. = 2.454; *P* = 0.002), SDA (regression coefficient = −1.091; S.E. = 0.450; *P* = 0.021), and DPA (regression coefficient = −3.639; S.E. = 1.569; *P* = 0.027).

**Table 4:** Adjusted regression coefficients (S.E.) for differences in omega-3 fatty acid relative to a one-unit increase in log10-transformed bis-(1-chloro-2-propyl) phosphate (BCPP), with results weighted for sampling strategy.

	**Eicosapentaenoic acid (μmol/L)**			**Docosahexaenoic acid (μmol/L)**			**Alpha-linolenic acid (μmol/L)**			**Stearidonic acid (μmol/L)**			**Docosapentaenoic acid (μmol/L)**		
	**Unweighted no./Population size**	**Regression coefficient (S.E.)**	***P***	**Unweighted no./Population size**	**Regression coefficient (S.E.)**	***P***	**Unweighted no./Population size**	**Regression coefficient (S.E.)**	***P***	**Unweighted no./Population size**	**Regression coefficient (S.E.)**	***P***	**Unweighted no./Population size**	**Regression coefficient (S.E.)**	***P***
Overall	1,210/ 53,466,400	−4.44 (2.28)	0.06	1,148/ 51,175,298	2.8 (4.57)	0.545	1,338 (57068532)	−5.58 (3.31)	0.102	1,127/ 49,353,600	−0.76 (0.27)	0.007	1,229/ 53,859,774	−3.02 (1.45)	0.045
**Sex**															
Male	578(25098217)	−0.67(3.81)	0.862	554(24390323)	0.98(6.17)	0.874	635(26652434)	−7.6(4.33)	0.089	541(23559140)	−0.82(0.34)	0.021	585(25284654)	−3.02(2.64)	0.261
Female	632(28361814)	−7.37(4.19)	0.088	584(26784975)	5.29(6.62)	0.43	703(30416098)	−3.77(3.87)	0.338	586(25794460)	−0.69(0.30)	0.028	644(28575120)	−2.88(1.57)	0.075
**Age (years)**															
20–50	628(28793617)	−8.22(2.45)	0.002	585(27142107)	−6.59(6.1)	0.288	688(30622159)	−10.13(5.09)	0.055	557(25985209)	−1.09(0.45)	0.021	636(29055012)	−3.64(1.57)	0.027
>50	582(24672783)	2.02(4.37)	0.647	563(24033191)	15.25(8.62)	0.086	650(26446373)	0.09(6.05)	0.988	570(23368390)	−0.36(0.43)	0.408	593(24804763)	−1.96(2.85)	0.496
**Body mass index (kg/m** ^**2**^ **)**															
<25	344(14680564)	2.06(4.36)	0.64	320(13835737)	10.92(8.1)	0.187	386(15584032)	−5.06(5.52)	0.366	309(13025788)	−1.1(0.61)	0.084	354(14845303)	0.06(1.75)	0.971
25–30	413(19028922)	−7.03(4.07)	0.094	393(18186051)	−6.44(8.75)	0.467	455(20343822)	−10.69(5.25)	0.05	391(17922549)	−0.85(0.41)	0.043	416(19124390)	−5.60(2.60)	0.039
≧30	446(19611553)	−6.36(6.53)	0.337	428(19008148)	8.45(0.33)	0.329	497(21140679)	−0.04(6.51)	0.996	427(18405263)	−0.38(0.37)	0.307	452(19744719)	−3.59(2.46)	0.154
**Alcohol consumption (drink/year)**															
<12	289(10184079)	4.99(5.78)	0.395	289(10184079)	7.2(12.68)	0.574	327(11054304)	−4.47(8.37)	0.597	292(9998263)	−0.21(0.34)	0.545	307(10654708)	−0.35(3.17)	0.913
≧12	862(41115945)	−6.1(2.8)	0.0362	859(40991219)	2.14(5.26)	0.688	913(42559157)	−7.08(4.07)	0.091	835(39355337)	−0.88(0.30)	0.006	865(41072743)	−3.81(1.69)	0.031
Income (USD)															
<45,000	604(20765108)	−8.72(4.38)	0.055	575(19834326)	1.57(6.88)	0.822	639(21772125)	−9.47(4.94)	0.064	534(18692240)	−0.77(0.42)	0.074	589(20351227)	−2.98(2)	0.146
≧45,000	606(32701293)	−2.22(2.93)	0.455	573(31340972)	2.83(6.72)	0.676	647(33725326)	−3.08(4.74)	0.52	547(29284015)	−0.76(0.41)	0.072	594(32097279)	−3.08(2.16)	0.163
Ethnicity															
Mexican-American	150(4404881)	4.33(6.35)	0.507	139(4046923)	16.56(11.01)	0.158	171(5006399)	−7.26(9.73)	0.469	139(4149747)	−0.11(0.64)	0.873	157(4702000)	−1.12(3.44)	0.75
Other Hispanic	110(2876210)	13.64(6.36)	0.049	104(2666953)	17.16(7.63)	0.041	139(3563313)	−1.17(8.45)	0.892	115(2761828)	−0.29(0.39)	0.472	126(3248913)	2.08(3.02)	0.502
Non-Hispanic White	562(38067694)	−6.81(2.84)	0.023	543(36842123)	4.74(5.90)	0.428	591(39536591)	−6.20(4.44)	0.173	530(35419924)	−1.02(0.35)	0.007	553(37780032)	−3.88(1.77)	0.036
Non-Hispanic Black	222(4371286)	−6.93(4.17)	0.112	213(4162272)	−16.31(9.05)	0.086	245(4804034)	−1.5(6.88)	0.83	204(3918381)	0.06(0.62)	0.927	222(4341893)	−4.27(1.76)	0.024
Other Race - Including Multi-Racial	166(3746329)	4.65(7.59)	0.546	149(3457027)	4.43(17.91)	0.807	192(4157195)	−3.30(7.95)	0.682	139(3103719)	0.26(0.51)	0.612	171(3786936)	0.12(3.72)	0.976

### Association between BCEP and DBUP exposure and the levels of omega-3 fatty acids

The adjusted regression coefficients (S.E.) for changes in EPA, DHA, ALN, SDA, and DPA with a one-unit rise in log-transformed BCEP and DBUP are detailed in Table 5 and Table 6. A one-unit increase in log BCEP levels was linked to a reduction in DHA (regression coefficient = −7.112; S.E. = 3.227; *P* = 0.035) across all participants. Subgroup analyses revealed a more pronounced decrease in SDA among men (regression coefficient = −1.096; S.E. = 0.508; *P* = 0.038) and younger adults (regression coefficient = −1.040; S.E. = 0.420; *P* = 0.019). Additionally, a one-unit rise in log DBUP levels was associated with a decrease in ALN (regression coefficient = −9.670; S.E. = 4.520; *P* = 0.04) and an increase in DPA (regression coefficient = 4.351; S.E. = 1.390; *P* = 0.004) across all participants. The effects of DBUP were found to be more significant on ALN, SDA, and DPA in older adults (>50 years).

**Table 5:** Adjusted regression coefficients (S.E.) for differences in omega-3 fatty acid relative to a one-unit increase in log10-transformed bis-2-chloroethyl phosphate (BCEP), with results weighted for sampling strategy.

	**Eicosapentaenoic acid (μmol/L)**			**Docosahexaenoic acid (μmol/L)**			**Alpha-linolenic acid (μmol/L)**			**Stearidonic acid (μmol/L)**			**Docosapentaenoic acid (μmol/L)**		
	**Unweighted no./Population size**	**Regression coefficient (S.E.)**	***P***	**Unweighted no./Population size**	**Regression coefficient (S.E.)**	***P***	**Unweighted no./Population size**	**Regression coefficient (S.E.)**	***P***	**Unweighted no./Population size**	**Regression coefficient (S.E.)**	***P***	**Unweighted no./Population size**	**Regression coefficient (S.E.)**	***P***
Overall	1,210/ 53,466,400	−3.86(3.29)	0.249	1,148/ 51,175,298	−7.11 (3.23)	0.035	1,338 (57068532)	−1.50 (3.09)	0.736	1,127/ 49,353,600	−0.46 (0.31)	0.15	1,229/ 53,859,774	−1.09 (1.38)	0.434
**Sex**															
Male	578(25098217)	−4.53(4.09)	0.276	554(24390323)	−8.73(5.89)	0.148	635(26652434)	−5.08(4.81)	0.3	541(23559140)	−1.1(0.51)	0.038	585(25284654)	−3.52(2.00)	0.089
Female	632(28368184)	−4.52(3.92)	0.257	594(26784975)	−5.59(5.18)	0.288	703(30416098)	2.21(2.65)	0.411	586(25794460)	−0.05(0.28)	0.863	644(28575120)	0.12(1.36)	0.929
Age (years)															
20–50	628(28793617)	−6.71(3.66)	0.076	585(27142107)	−4.38(5.38)	0.422	688(30622159)	−4.32(4.80)	0.376	557(25985209)	−1.04(0.42)	0.019	636(29055012)	−1.26(1.38)	0.37
>50	582(24672783)	−1.98(4.42)	0.657	563(24033191)	−10.85(6.04)	0.082	650(26446373)	1.68(3.64)	0.648	570(23368390)	0.11(0.36)	0.772	593(24804763)	−0.92(2.22)	0.682
**Body mass index (kg/m** ^**2**^ **)**															
<25	344(14680564)	2.84(4.64)	0.545	320(13835737)	−3.05(5.89)	0.608	386(15584032)	−2.21(5.66)	0.699	309(13025788)	−0.36(1.10)	0.746	354(14845303)	0.79(2.46)	0.751
25–30	413(19028922)	−6.17(3.61)	0.098	393(18186051)	−7.14(6.35)	0.269	455(20343822)	−0.82(3.87)	0.833	391(17922549)	−0.45(0.44)	0.306	416(19124390)	−1.86(1.9)	0.335
≧30	446(19611553)	−6.56(5.16)	0.213	428(19008418)	−8.79(4.53)	0.061	497(21140679)	−0.59(5.21)	0.91	427(18495263)	−0.54(0.31)	0.089	452(19744719)	−2.34(1.38)	0.099
**Alcohol consumption (drink/year)**															
<12	289(10184079)	−2.42(6.08)	0.694	289(10184079)	−3.41(11.27)	0.764	327(11054304)	6.08(5.62)	0.288	292(9998263)	−0.32(0.21)	0.133	307(10654708)	1.23(2.89)	0.673
≧12	862(41115945)	−4.59(3.9)	0.248	859(40991219)	−8.00(4.13)	0.061	913(42559157)	−2.88(3.8)	0.454	835(39355337)	−0.51(0.4)	0.211	865(41072743)	−1.98(1.71)	0.256
Income (USD)															
<45,000	604(20765108)	−7.66(4.64)	0.109	575(19834326)	−10.3(4.59)	0.032	639(21772125)	−3.48(3.49)	0.326	534(18692240)	- 0.66(0.42)	0.128	589(20351227)	−1.46(1.61)	0.369
> = 45,000	606(32701293)	−1.68(3.91)	0.67	573(31340972)	−4.72(4.56)	0.309	647(33725326)	1.75(5.34)	0.745	547(29285015)	−0.29(0.5)	0.57	594(32097279)	−0.77(1.97)	0.697
**Ethnicity**															
Mexican-American	150(4404881)	3.47(3.02)	0.271	139(4046923)	−13.87(6.41)	0.051	171(5006399)	−2.4(4.90)	0.633	139(4149747)	−0.20(0.36)	0.583	157(4702000)	0.86(1.79)	0.639
Other Hispanic	110(2876210)	3.24(3.57)	0.379	104(2666953)	−0.33(7.88)	0.968	139(3563313)	11.13(7.27)	0.145	115(02761828)	0.20(0.43)	0.65	126(3248913)	4.38(2.54)	0.105
Non-Hispanic White	562(38067694)	−4.88(4.33)	0.269	543(36842123)	−6.67(3.96)	0.103	591(39536590)	−1.49(4.74)	0.756	530(35419924)	−0.57(0.46)	0.217	553(37780032)	1.85(1.87)	0.331
Non-Hispanic Black	222(4371286)	−6.68(4.5)	0.153	213(4162272)	−9.80(10.11)	0.343	245(4805034)	−3.06(3.63)	0.408	204(3918381)	−0.44(0.24)	0.077	222(4341893)	−2.16(2.15)	0.328
Other Race - Including Multi-Racial	166(3746329)	−6.19(7.75)	0.433	149(3457027)	13.72(12.48)	0.284	192(4157194)	−1.69(6.16)	0.786	139(3103719)	0.18(0.49)	0.724	171(3786936)	−0.37(2.73)	0.894

**Table 6:** Adjusted regression coefficients (S.E.) for differences in omega-3 fatty acids relative to a one-unit increase in log10-transformed dibutyl phosphate (DBUP), with results weighted for sampling strategy.

	**Eicosapentaenoic acid (μmol/L)**			**Docosahexaenoic acid (μmol/L)**			**Alpha-linolenic acid (μmol/L)**			**Stearidonic acid (μmol/L)**			**Docosapentaenoic acid (μmol/L)**		
	**Unweighted no./Population size**	**Regression coefficient (S.E.)**	***P***	**Unweighted no./Population size**	**Regression coefficient (S.E.)**	***P***	**Unweighted no./Population size**	**Regression coefficient (S.E.)**	***P***	**Unweighted no./Population size**	**Regression coefficient (S.E.)**	***P***	**Unweighted no./Population size**	**Regression coefficient (S.E.)**	***P***
Overall	1,210/ 53,466,400	−8.07 (4.16)	0.061	1,148/ 51,175,298	−9.32 (6.1)	0.137	1,338 (57068532)	−9.67 (4.52)	0.04	1,127/ 49,353,600	−0.78 (0.43)	0.078	1,229/ 53,859,774	−4.35 (1.39)	0.004
**Sex**															
Male	578(25098217)	−8.81(5.05)	0.091	554(24390323)	−17.40(8.01)	0.037	635(26652434)	−9.88(6.39)	0.132	541(23559140)	−1.13(0.76)	0,147	585(25284654)	−5.18(1.91)	0.011
Female	632(28368184)	−6.38(5.99)	0.295	594(26784975)	−0.24(9.05)	0.979	703(30416098)	−9.38(5.38)	0.091	586(25794460)	−0.43(0.44)	0.332	644(28575120)	−3.41(1.41)	0.021
**Age (years)**															
20–50	628(28793617)	−7.35(5.49)	0.19	585(247142107)	−15.95(7.90)	0.052	688(3.0622159)	−4.93(6.42)	0.448	557(25985209)	−0.41(0.7)	0.564	636(29055012)	−1.62(2.26)	0.479
>50	582(24672783)	−6.75(6.43)	0.301	563(24033191)	−0.63(9.71)	0.949	650(26446373)	−14.69(6.17)	0.023	570(23368390)	−1.14(0.36)	0.004	593(24804763)	−6.8(1.81)	0.001
**Body mass index (kg/m** ^**2**^ **)**															
<25	344(14680564)	−0.18(8.12)	0.983	320(13835737)	1.82(11.01)	0.87	386(15584032)	0.34(9.2)	0.971	309(13025788)	0.51(1.17)	0.666	354(14845303)	0.56(2.94)	0.849
25–30	413(19028922)	−15.97(6.99)	0.029	393(18186051)	−32.72(11.08)	0.006	455(20343822)	−10.68(6.04)	0.086	391(17922549)	−0.86(0.53)	0.112	416(19124390)	−6.12(3.08)	0.056
≧30	446(19611553)	−8.71(4.11)	0.042	428(19008148)	3.93(8.44)	0.644	497(21140679)	−15.81(9.72)	0.114	427(18405263)	−1.55(0.7)	0.033	452(19744719)	−6.16(2.01)	0.004
**Alcohol consumption (drink/year)**															
<12	289(10184079)	0.73(7.34)	0.921	289(10184079)	−3.56(14.96)	0.814	327(11054304)	−15.38(7.96)	0.063	292(9998263)	- 0.63(0.37)	0.096	307(10654708)	−4.29(2.48)	0.093
≧12	862(41115945)	−10.00(5.17)	0.062	859(40991219)	−10.49(8.03)	0.201	913(42559157)	−10.86(6.02)	0.081	835(39355336)	−0.84(0.55)	0.133	865(41072743)	−5.11(1.90)	0.011
**Income (USD)**															
<45,000	604(20765108)	−3.30(5.71)	0.567	575(19834326)	−4.25(8.61)	0.625	639(21772125)	−23.41(7.14)	0.003	534(18692240)	−1.73(0.89)	0.06	589(20351227)	−4.55(2.36)	0.062
≧45,000	606(32701293)	−11.02(6.21)	0.085	573(31340972)	−12.97(9.73)	0.192	647(33725326)	−0.55(6.18)	0.929	547(29284015)	−0.12(0.54)	0.831	594(32097279)	−3.87(2.4)	0.117
**Ethnicity**															
Mexican-American	150(4404881)	3.69(5.61)	0.522	139(4046923)	−17.89(6.12)	0.013	171(5006399)	−12.01(11.95)	0.333	139(4149747)	−0.19(0.60)	0.754	157(4702000)	−2.86(3.32)	0.404
Other Hispanic	110(2876210)	14.27(7.94)	0.094	104(2666953)	3.87(14.77)	0.797	139(3563313)	−5.31(6.57)	0.431	115(2761828)	−0.73(0.57)	0.22	126(3248913)	4.94(4.75)	0.315
Non-Hispanic White	562(38067694)	−10.60(4.68)	0.031	543(36842123)	−9.73(8.25)	0.247	591(39536591)	−10.47(5.78)	0.08	530(35419924)	−0.88(0.58)	0.143	553(37780032)	−5.5(1.80)	0.005
Non-Hispanic Black	222(4371286)	−11.07(6.08)	0.083	213(4162272)	−10.43(12.02)	0.395	245(4805034)	−9.44(5.55)	0.103	204(3918381)	−0.67(0.28)	0.029	222(4341893)	−4.64(2.41)	0.068
Other Race - Including Multi-Racial	166(3746329)	−4.87(11.09)	0.665	149(3457027)	0.49(13.78)	0.972	192(4157195)	−4.37(10.58)	0.683	139(3103719)	−0.21(0.58)	0.721	171(3786936)	−2.40(3.90)	0.544

### Correlations between the quartiles of OPFRs and omega-3 fatty acids

**Fig. 2:**
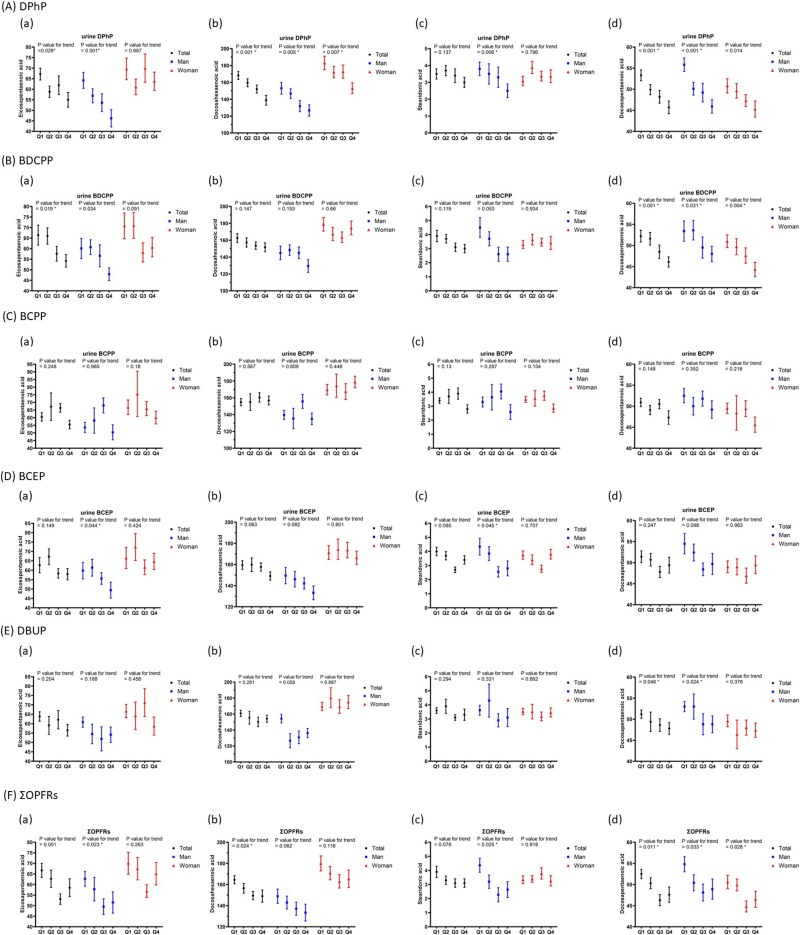
Mean and SE of eicosapentaenoic acid, docosahexaenoic acid, stearidonic acid, and docosapentaenoic acid across quartiles of OPFRs in linear regression models, with results weighted for sampling strategy. A) Diphenyl phosphate (DPhP), B) bis(1,3-dichloro-2-propyl) phosphate (BDCPP), C) bis(1-chloro-2-propyl) phosphate (BCPP). Mean and SE of eicosapentaenoic acid, docosahexaenoic acid, stearidonic acid, and docosapentaenoic acid across quartiles of OPFRs in linear regression models, with results weighted for sampling strategy. D) bis(2-chloroethyl) phosphate (BCEP), E) di-n-butyl phosphate (DnBP), and sum of OPFRs (ΣOPFRs).

After adjusting for covariates in the multiple regression analysis, Fig. 2 illustrates the associations between OPFR quartiles and the levels of EPA, DHA, and DPA among participants. Supplementary Table 2 presents the exact mean values and standard errors of each omega-3 fatty acid across the quartiles of individual OPFRs. As urine DPhP levels increased across quartiles, mean EPA (p for trend = 0.028), DHA (p for trend <0.001), and DPA (p for trend <0.001) levels significantly dropped in all participants. The differences between the highest and lowest DPhP quartiles were 18.2, 17.5, and 14.3% for EPA, DHA, and DPA, respectively.

Similarly, increasing BDCPP quartiles were associated with significant reductions in EPA (p for trend = 0.019) and DPA (p for trend = 0.001) levels in all participants. EPA levels dropped by 18.4% in all participants when comparing the highest to the lowest quartiles, while DPA levels declined by 11.7% in all participants. For DBUP, increasing quartiles were linked to a significant reduction in DPA levels (p for trend = 0.046). Additionally, higher total OPFRs (ΣOPFRs) were associated with a significant decrease in DHA (p for trend = 0.024) and DPA (p for trend = 0.011), with DHA and DPA levels dropping by 9.4 and 9.3% in the highest quartile compared to the lowest.

## Discussion

In this study, we found a statistically significant correlation between five OPFRs and serum omega-3 fatty acid levels. After accounting for confounding factors, we observed that DPhP levels had a negative correlation with EPA, DHA, SDA, and DPA levels; BDCPP levels were negatively related to EPA levels; BCPP levels were negatively associated with SDA and DPA levels; BCEP levels were negatively correlated with DHA levels; and DBUP levels showed a negative association with ALN and DPA levels. Increases in quartile levels of DPhP and BDCPP were negatively correlated with EPA, with differences of 18.2 and 18.4% between the highest and lowest quartiles, respectively. Furthermore, quartile increases in DPhP and ΣOPFRs were negatively correlated with DHA levels, with differences of 17.5 and 9.4% between the upper and lower quartiles, respectively. DPhP, BDCPP, and ΣOPFRs exhibited negative correlations with DPA levels.

Omega-3 PUFAs have demonstrated anti-inflammatory, antihypertensive, and triglyceride-lowering effects,^19^^,^^20^ which contribute to reduced cardiovascular risk^14^^,^^21^ Omega-3 PUFAs inhibit the transcription of sterol regulatory element-binding protein (SREBP) genes, which reduces the de novo synthesis of fatty acids and triglycerides. Additionally, they promote fatty acid oxidation and triglyceride catabolism in adipose and muscle tissues, while also enhancing the clearance of triglyceride-rich lipoproteins by modulating the activity of peroxisome proliferator-activated receptor (PPAR) genes.^22^ Meta-analyses and clinical trials, including The Reduction of Cardiovascular Events with Icosapent Ethyl–Intervention trial (REDUCE-IT), have linked higher omega-3 intake—particularly EPA, DHA, and DPA—to reduced risks of cardiovascular events and all-cause mortality^14^^,^^21^^,^^23^^,^^24^ Emerging evidence also suggests that OPFR exposure negatively impacts metabolic health. Prior studies have associated urinary OPFR metabolites with elevated BMI, triglyceride levels, and decreased HDL-C concentrations.^11^^,^^12^ Our findings are consistent with these observations, showing that exposure to DPhP and BDCPP is associated with lower EPA and DHA levels. Additionally, experimental studies indicate that OPFRs such as TCEP and TBEP may induce oxidative stress, cardiac fibrosis, and reproductive toxicity^8^^,^^25–27^ These results highlight the potential metabolic, cardiovascular, and reproductive health risks associated with OPFR exposure.

The distribution and types of OPFRs vary globally based on regional regulatory environments, industrial applications, and market demand. For instance, TCPP is frequently detected in the surface waters of Australia, France, Germany, and the United States. TBEP is often found in the surface water of countries such as Australia, Italy, and Spain. In China, TCEP is one of the most detected OPFRs in surface waters.^27^ Currently, monitoring human exposure to OPFRs primarily involves measuring the concentrations of OPFRs and their metabolites in urine. Guo et al. analyzed blood and morning urine samples from 120 volunteers in Chengdu and reported that EHDPP, Tris(chloropropyl) phosphate (TCIPP), and TPhP were the most common OPFRs in blood, whereas BCEP, BDCPP, DBUP, and DPhP were the predominant metabolites in urine.^28^ The study enrolled 391 volunteers in Taiwan and found that DPhP was detected in approximately 79.5% of urine samples, followed by tri(n-butyl)phosphate (TNBP) (79.2%) and TBEP (54.6%) in adults.^29^ According to 2013–2014 NHANES in United States, BDCPP and DPhP were detected in approximately 92% of urine specimen of study participants, while BCEP, DBUP and BCPP were detected in 89, 81, and 61% of the study participants, respectively.^4^ Urinary OPFR metabolite concentrations were standardized by both volume (μg/L) and creatinine-adjusted values (μg/g creatinine). This dual standardization allows for comparisons that account for urine dilution effects. The geometric means (GMs) and percentiles were calculated for both types of measurements where the detection frequency exceeded 60%. Additionally, since these metabolites have the highest detection rates (>50%) in the NHANES dataset, our study specifically focused on these metabolites to explore and analyze their relationships with PUFA levels. We discovered that DPhP and BDCPP are associated with a decrease in EPA, and DPhP is associated with a decrease in both ΣOPFRs and DHA from the 2013–2014 NHANES database. Studies about the effects and mechanism of lipid metabolism related to OPFRs exposure in human are needed for elucidation, besides, the connections between omega-3 fatty acid levels and OPFRs such as TBEP which were not available in the NHANES database should be evaluated in the future since the predominant OPFRs in human samples varied between different areas.

Dietary habits, lifestyle factors, and physiological characteristics play significant roles in influencing both omega-3 fatty acid and OPFR levels in humans. Dietary habits are a primary determinant of omega-3 levels, as highlighted by Pizzini et al., who emphasized the role of omega-3 intake in lipid metabolism and reverse cholesterol transport.^30^ Simultaneously, OPFR exposure is significantly affected by various factors, including age, sex, and environmental behaviors, with younger individuals and those in certain occupations experiencing higher exposure levels.^29^ Additionally, another study demonstrated that specific lifestyle choices, such as occupational exposures and alcohol drinking, can modify OPFR levels and their interactions with metabolic pathways.^31^ These findings underscore the complex interplay between dietary, behavioral, and environmental factors in determining omega-3 and OPFR levels, warranting further investigation into their combined effects.

Several recent studies have shown that OPFR exposure may affect lipid metabolism. A study in mice found that triphenyl phosphate (TPP) inhibited a specific subset of liver CEs, leading to elevated levels of low-density lipoprotein C (LDL-C) and VLDL in the blood. This inhibition also causes serum hypertriglyceridemia and disrupts lipoprotein profiles.^32^ Another study using RAW264.7 murine macrophage cells revealed that TPP induced ER stress and enhanced the inflammatory response. This subsequently led to the downregulation of lysophosphatidylcholine acyltransferase 3 (Lpcat3), thereby decreasing fatty acid saturation.^9^ Peroxisome proliferator-activated receptors (PPARs) are transcription factors that are essential for regulating lipid metabolism. PPARγ promotes the absorption of glucose and dietary fatty acids during the fed state, along with lipogenesis, triglyceride synthesis, and storage.^33^ Du et al. found that exposure to TPP significantly activated the PPARs signaling pathway and disturbed lipid metabolism in the liver of zebrafish.^34^ Perinatal exposure to TPP activated PPARγ as a molecular initial event, and induced key processes including lipid accumulation, disturbed hormone secretion, impaired placental angiogenesis, and abnormal cell apoptosis in mice.^35^ However, most of these studies have been limited to animal experiments or cell line research, and studies on the disruption of lipid metabolism by OPFRs in humans are limited. Previous research utilizing the NHANES database revealed that BCEP and BDCPP were associated with higher BMI, and that DPhP was positively correlated with triglyceride levels.^11^ Zhao et al. reported that exposure to EHDPP and TPP was associated with increased human total cholesterol (TC) and triglyceride (TG) levels in humans.^36^ Another study utilized a dataset from the 2013–2014 NHANES dataset and reported that DPhP, BDCPP, and BCEP levels were negatively correlated with high-density lipoprotein C (HDL-C) concentration, whereas DPhP and BCPP levels were negatively associated with TC levels.^12^ Our study found that DPhP and BDCPP were associated with a decrease in EPA, and DPhP is associated with a decrease in both ΣOPFRs and DHA. Previous studies have reported that OPFR exposure disrupts TG metabolism. Our findings suggest that OPFR exposure is associated with a reduction in omega-3 fatty acid levels, implying that OPFRs may affect fatty acid metabolism, and subsequently interfere with TG levels and lipogenesis.

OPFRs are emerging flame retardants that can reduce the flammability of consumer products and building materials. Owing to the persistence, bioaccumulation, and toxicity of PBDEs in the environment, they have gradually been phased out of the flame-retardant market. Consequently, other low-cost and effective flame retardants have replaced these, with OPFRs being the most popular. Since 2007, OPFR consumption in China has increased rapidly at a growth rate of 15% each year.^37^ OPFRs typically metabolize faster and have shorter half-lives than previously used flame retardants, such as PBDEs. Owing to their rapid breakdown and widespread dispersion in the environment, human exposure to OPFRs tends to be continuous, resulting in a phenomenon known as “pseudo-persistence.” Due to being physically bound additives, OPFRs can easily detach from products during use and be released into the environment. Recent studies have detected OPFRs in various environments and organisms, including the air,^38^ soil,^39^ sea water,^27^ fish,^40^ and even human breast milk.^3^ Humans are exposed to OPFRs via skin contact, inhalation, and ingestion.^41^ Daily exposure to OPFRs is estimated to be 149 ng/kg of body weight per day through air, 279 ng/kg of body weight per day through skin contact, and 390 ng/kg of body weight per day through dust ingestion. This highlights the fact that dust ingestion is a major source of indoor OPFR exposure.^42^ In China, the estimated daily intakes of OPFRs by dust ingestion for adults and children were 2.12 and 11.06 ng/kg of body weight per day (average), respectively.^43^ Children have greater exposure to OPFRs through dust ingestion than adults because of their lower body weight and more frequent hand-to-mouth behavior.^44^ The use of OPFRs is becoming more prevalent, and mounting evidence points to their potential health hazards, including neurotoxicity, damage to reproductive function, endocrine disruption, developmental toxicity, and carcinogenicity.^7^^,^^45^^,^^46^ Our research indicates that exposure to OPFRs may be associated with reduced levels of omega-3 fatty acids, which may lead to adverse health effects. Therefore, it is crucial to focus on the future use of OPFRs.

## Conclusion

In the present study, we observed a statistically significant associations between OPFRs and serum omega-3 fatty acid concentration. After adjusting for confounding factors, the DPhP level was negatively associated with EPA, DHA, SDA, and DPA levels, the BDCPP level was negatively associated with EPA levels, especially in men. Compared to the lowest quartiles of DPhP and BDCPP, the highest quartile showed a decrease in EPA by approximately 18.2 and 18.4%, respectively. Additionally, quartile increases in the levels of DPhP and ΣOPFRs were negatively correlated with DHA levels, with differences of approximately 17.5 and 9.4% between the upper and lower quartiles, respectively.

The results underscore the need for further research to elucidate the underlying biological mechanisms and to confirm these associations in controlled experimental or longitudinal studies. Additionally, future investigations should aim to address the broader spectrum of OPFRs and other potential environmental exposures to better understand their impact on lipid metabolism. By highlighting these preliminary associations, this study serves as a foundation for generating hypotheses and guiding future research in this field.

### Limitations

First, the data for this study were derived from the U.S. NHANES database, and exposure levels may differ in other countries or regions, which could lead to variations in results. Second, this study only collected exposure data for OPFRs, whereas many environmental toxins can affect omega-3 fatty acids. Thus, the influence of other environmental endocrine disruptors cannot be completely ruled out. Third, the NHANES database includes only a limited number of OPFRs, whereas more than 20 types have been detected in the environment. Additionally, a proportion of participants had OPFR concentrations below the LODs, which may have affected the outcomes of the linear regression models. Fourth, although major covariates such as age, sex, ethnicity, income, alcohol consumption, BMI, and total polyunsaturated fatty acid intake were adjusted for, other potential factors that could influence circulating omega-3 fatty acid levels, such as seafood consumption, physical activity, albuminuria, and creatinine clearance, were not included in the dataset and thus could have influenced the results. Fifth, the OPFRs not tested in this study may also be related to omega-3 fatty acid levels; therefore, our findings may not represent the full spectrum of OPFRs. Finally, this study is observational in nature and relies on correlational analysis, which limits our ability to establish causality.

## Supplementary Material

supplementary_figure_1_tfaf119

supplementary_table_tfaf119

## Data Availability

The data used in this study were obtained from the 2011–2014 National Health and Nutrition Examination Survey (NHANES) dataset, which is publicly available on the NHANES website.
