# Supplementary figures and images for: The association of organophosphate flame retardants (OPFRs) exposure on omega-3 fatty acids metabolism: evidence derived from the United States general population
Source: Toxicol Res (Camb). 2025 Aug 17;14(4):tfaf119. doi: 10.1093/toxres/tfaf119 (PMC12358045; doi:10.1093/toxres/tfaf119)

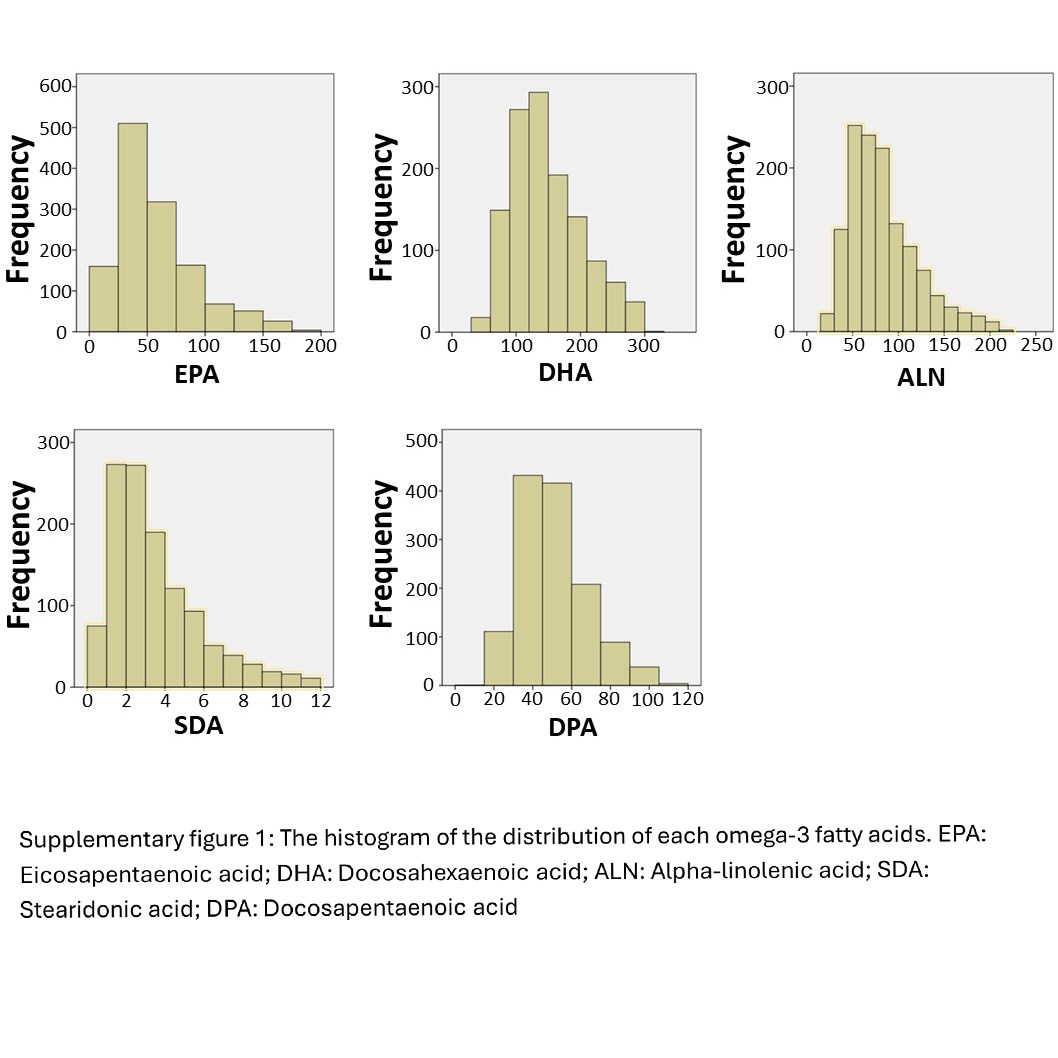

Supplement: supplementary_figure_1_tfaf119 [file supplementary_figure_1_tfaf119.jpeg]
