# Supplementary material for: The association of organophosphate flame retardants (OPFRs) exposure on omega-3 fatty acids metabolism: evidence derived from the United States general population
Source: Toxicol Res (Camb). 2025 Aug 17;14(4):tfaf119. doi: 10.1093/toxres/tfaf119 (PMC12358045; doi:10.1093/toxres/tfaf119)
Supplement: supplementary_table_tfaf119 [file supplementary_table_tfaf119.docx]

**Supplementary Table 1** detection frequency of major organophosphate flame retardant metabolites in included participants

| Number = 1350 (%) | Under detection limits | Detection frequency (%) |
| --- | --- | --- |
| diphenyl phosphate (DPhP) | 69 | 94.9 |
| bis(1,3-dichloro-2-propyl) phosphate (BDCPP) | 42 | 96.9 |
| bis-(1-chloro-2-propyl) phosphate (BCPP) | 261 | 80.7 |
| bis-2-chloroethyl phosphate (BCEP) | 90 | 93.3 |
| dibutyl phosphate (DBUP) | 159 | 88.2 |

Supplementary table 2 Mean and SE of omega-3 fatty acids, across quartiles of OPFRs in linear regression models, with results weighted for sampling strategy.

|  | Eicosapentaenoic acid (mean, SE) | |  | Docosahexaenoic acid (mean, SE) | | | Alpha-linolenic acid (mean, SE) | | | Stearidonic acid (mean, SE) | | | Docosapentaenoic acid (mean, SE) | | |
| --- | --- | --- | --- | --- | --- | --- | --- | --- | --- | --- | --- | --- | --- | --- | --- |
| **urine DPhP** | Total | Man | Woman | Total | Man | Woman | Total | Man | Woman | Total | Man | Woman | Total | Man | Woman |
| Q1 | 67.2 (2.8) | 64.2 (3.8) | 69.6 (5.1) | 168.2 (4.8) | 153.0 (7.0) | 183.0 (8.0) | 92.4 (3.2) | 92.3 (3.9) | 92.1 (5.1) | 3.5 (0.3) | 3.8 (0.4) | 3.08 (0.27) | 53.3 (1.3) | 55.8 (1.6) | 50.8 (1.7) |
| Q2 | 58.8 (2.7) | 56.9 (3.3) | 61.1 (3.7) | 159.5 (4.7) | 146.7 (5.7) | 172.2 (6.7) | 92.3 (3.5) | 92.4 (6.2) | 92.0 (3.8) | 3.7 (0.3) | 3.5 (0.6) | 3.88 (0.36) | 49.9 (1.2) | 50.1 (1.5) | 49.6 (1.7) |
| Q3 | 61.9 (4.4) | 53.6 (4.2) | 70.0 (6.7) | 152.0 (4.7) | 131.8 (6.6) | 172.7 (7.8) | 85.9 (4.1) | 82.4 (5.0) | 89.5 (4.2) | 3.4 (0.4) | 3.3 (0.6) | 3.37 (0.29) | 48.2 (1.5) | 49.2 (2.2) | 47.3 (1.4) |
| Q4 | 55.0 (3.5) | 46.2 (4.1) | 63.8 (4.3) | 138.8 (5.8) | 126.7 (6.7) | 153.1 (6.2) | 85.9 (3.2) | 86.3 (5.8) | 86.0 (3.9) | 3.0 (0.3) | 2.5 (0.4) | 3.36 (0.38) | 45.7 (1.5) | 45.9 (1.6) | 45.3 (1.9) |
| P value for trend | 0.028 | 0.001 | 0.687 | <0.001 | 0.005 | 0.007 | 0.091 | 0.116 | 0.32 | 0.137 | 0.006 | 0.796 | <0.001 | <0.001 | 0.014 |
| **urine BDCPP** | Total | Man | Woman | Total | Man | Woman | Total | Man | Woman | Total | Man | Woman | Total | Man | Woman |
| Q1 | 66.4 (4.7) | 60.1 (4.8) | 70.8 (6.1) | 162.6 (5.2) | 144.9 (8.0) | 178.8 (8.0) | 92.9 (3.8) | 97.0 (7.3) | 89.6 (3.6) | 3.9 (0.4) | 4.5 (0.7) | 3.29 (0.28) | 52.2 (1.4) | 53.4 (2.4) | 51.0 (1.5) |
| Q2 | 66.0 (3.9) | 60.7 (3.5) | 71.0 (6.1) | 157.3 (5.5) | 148.3 (6.3) | 167.3 (7.8) | 90.6 (3.0) | 88.6 (4.3) | 92.8 (4.1) | 3.7 (0.3) | 3.7 (0.5) | 3.64 (0.38) | 51.6 (1.5) | 53.6 (2.3) | 49.7 (1.9) |
| Q3 | 57.6 (3.5) | 56.6 (5.2) | 58.2 (4.5) | 153.5 (4.3) | 145.0 (6.9) | 163.5 (6.3) | 86.7 (5.3) | 87.9 (7.2) | 85.3 (4.2) | 3.1 (0.3) | 2.6 (0.5) | 3.46 (0.28) | 48.5 (1.6) | 49.5 (2.5) | 47.6 (1.8) |
| Q4 | 54.2 (3.0) | 47.9 (3.0) | 60.7 (4.6) | 151.6 (5.3) | 129.4 (7.5) | 174.6 (8.1) | 87.7 (4.4) | 82.2 (5.0) | 94.5 (7.3) | 3.0 (0.3) | 2.6 (0.5) | 3.40 (0.46) | 46.1 (1.2) | 48.0 (1.8) | 44.3 (1.7) |
| P value for trend | 0.019 | 0.034 | 0.091 | 0.147 | 0.153 | 0.66 | 0.36 | 0.17 | 0.764 | 0.119 | 0.053 | 0.934 | 0.001 | 0.031 | 0.004 |
| **urine BCPP** | Total | Man | Woman | Total | Man | Woman | Total | Man | Woman | Total | Man | Woman | Total | Man | Woman |
| Q1 | 60.5 (2.8) | 53.6 (3.3) | 66.8 (4.8) | 154.8 (4.3) | 139.6 (5.3) | 169.7 (6.2) | 89.9 (2.4) | 91.3 (3.4) | 89.1 (3.2) | 3.4 (0.2) | 3.3 (0.35) | 3.49 (0.22) | 50.9 (1.0) | 52.5 (1.7) | 49.5 (1.2) |
| Q2 | 67.2 (9.0) | 58.1 (8.3) | 75.5 (14.8) | 154.9 (9.9) | 135.3 (12.1) | 174.4 (13.9) | 90.5 (3.6) | 90.3 (8.2) | 90.3 (5.4) | 3.7 (0.5) | 3.64 (0.91) | 3.56 (0.55) | 49.1 (1.1) | 50.0 (2.1) | 48.4 (4.1) |
| Q3 | 66.4 (2.8) | 68.0 (4.9) | 65.9 (4.6) | 160.4 (5.6) | 155.7 (8.4) | 167.4 (9.4) | 92.3 (4.2) | 90.9 (6.1) | 93.2 (5.8) | 3.9 (0.4) | 4.05 (0.53) | 3.75 (0.33) | 50.5 (1.1) | 51.8 (1.8) | 49.4 (1.9) |
| Q4 | 55.5 (2.8) | 50.4 (4.9) | 60.1 (4.2) | 156.8 (5.3) | 134.9 (6.9) | 178.8 (6.9) | 86.1 (4.1) | 83.0 (4.7) | 89.1 (4.9) | 2.8 (0.3) | 2.58 (0.53) | 2.86 (0.29) | 47.3 (1.6) | 49.2 (2.1) | 45.6 (1.8) |
| P value for trend | 0.248 | 0.985 | 0.18 | 0.567 | 0.809 | 0.448 | 0.594 | 0.249 | 0.909 | 0.13 | 0.287 | 0.154 | 0.148 | 0.352 | 0.218 |
| **urine BCEP** | Total | Man | Woman | Total | Man | Woman | Total | Man | Woman | Total | Man | Woman | Total | Man | Woman |
| Q1 | 62.7 (4.1) | 59.8 (4.4) | 66.5 (5.6) | 159.5 (4.1) | 149.5 (7.7) | 171.3 (6.5) | 89.5 (4.1) | 92.1 (6.3) | 88.1 (3.7) | 4.0 (0.3) | 4.34 (0.59) | 3.75 (0.29) | 51.4 (1.4) | 54.5 (2.4) | 49.0 (1.4) |
| Q2 | 67.2 (4.2) | 61.4 (4.5) | 72.4 (7.2) | 159.8 (6.5) | 146.1 (7.3) | 174.4 (9.7) | 85.5 (3.3) | 84.7 (4.0) | 86.6 (5.0) | 3.7 (0.3) | 3.85 (0.49) | 3.41 (0.35) | 50.7 (1.5) | 52.4 (2.0) | 49.0 (1.9) |
| Q3 | 58.3 (2.5) | 55.6 (3.1) | 61.6 (3.9) | 157.6 (3.9) | 142.1 (5.3) | 173.7 (7.3) | 94.7 (6.1) | 94.2 (8.0) | 95.2 (5.4) | 2.7 (0.2) | 2.56 (0.37) | 2.77 (0.25) | 47.8 (1.3) | 48.4 (1.5) | 46.9 (1.8) |
| Q4 | 57.9 (3.0) | 49.4 (4.2) | 64.7 (4.3) | 149.1 (3.9) | 133.2 (6.5) | 166.4 (6.3) | 88.8 (3.3) | 85.4 (5.0) | 91.4 (4.6) | 3.4 (0.3) | 2.80 (0.53) | 3.81 (0.34) | 49.4 (1.9) | 49.7 (2.5) | 49.5 (2.1) |
| P value for trend | 0.149 | 0.044 | 0.424 | 0.063 | 0.082 | 0.601 | 0.688 | 0.72 | 0.238 | 0.095 | 0.045 | 0.707 | 0.247 | 0.098 | 0.963 |
| **urine DBUP** | Total | Man | Woman | Total | Man | Woman | Total | Man | Woman | Total | Man | Woman | Total | Man | Woman |
| Q1 | 63.8 (2.5) | 60.8 (2.7) | 66.7 (3.5) | 160.8 (3.9) | 154.2 (5.2) | 169.7 (5.1) | 92.3 (2.8) | 92.6 (4.7) | 92.1 (2.8) | 3.6 (0.2) | 3.62 (0.35) | 3.53 (0.23) | 51.2 (1.0) | 53.0 (1.2) | 49.6 (1.4) |
| Q2 | 59.1 (4.8) | 54.5 (5.2) | 64.2 (7.3) | 155.4 (8.0) | 126.7 (8.9) | 180.5 (12.5) | 94.3 (4.4) | 91.8 (9.1) | 96.0 (5.8) | 3.9 (0.5) | 4.30 (1.17) | 3.52 (0.52) | 49.4 (2.3) | 53.0 (3.0) | 46.4 (3.4) |
| Q3 | 62.1 (4.9) | 51.9 (6.4) | 71.2 (7.4) | 150.2 (5.9) | 130.8 (7.9) | 169.5 (8.5) | 86.9 (3.9) | 84.4 (5.8) | 88.7 (5.5) | 3.1 (0.2) | 2.90 (0.47) | 3.18 (0.31) | 48.6 (1.5) | 48.8 (2.5) | 48.0 (1.8) |
| Q4 | 56.5 (3.1) | 54.1 (4.1) | 58.7 (4.8) | 154.2 (4.5) | 136.3 (5.7) | 175.1 (8.2) | 84.5 (3.6) | 85.4 (5.6) | 84.4 (3.5) | 3.3 (0.4) | 3.10 (0.64) | 3.46 (0.32) | 47.8 (1.4) | 48.8 (2.0) | 47.4 (1.7) |
| P value for trend | 0.204 | 0.188 | 0.456 | 0.281 | 0.058 | 0.887 | 0.043 | 0.249 | 0.014 | 0.294 | 0.331 | 0.662 | 0.046 | 0.024 | 0.376 |
| **ΣOPFRs** | Total | Man | Woman | Total | Man | Woman | Total | Man | Woman | Total | Man | Woman | Total | Man | Woman |
| Q1 | 66.7 (3.3) | 62.7 (3.5) | 70.1 (5.2) | 164.5 (3.8) | 148.9 (6.7) | 180.0 (7.0) | 92.7 (3.1) | 94.1 (4.6) | 91.6 (4.0) | 3.9 (0.4) | 4.36 (0.49) | 3.34 (0.27) | 52.5 (1.1) | 54.8 (1.8) | 50.6 (1.6) |
| Q2 | 62.7 (4.1) | 57.8 (5.6) | 67.5 (5.3) | 156.5 (4.6) | 142.9 (6.1) | 170.8 (6.3) | 89.8 (4.6) | 90.1 (6.0) | 89.3 (4.1) | 3.3 (0.3) | 3.20 (0.47) | 3.39 (0.24) | 50.3 (1.3) | 50.4 (1.9) | 49.9 (1.4) |
| Q3 | 53.1 (2.5) | 49.5 (3.7) | 56.9 (3.0) | 149.5 (4.0) | 137.1 (6.2) | 163.0 (6.3) | 84.2 (2.6) | 81.8 (4.1) | 87.6 (3.3) | 3.1 (0.3) | 2.27 (0.50) | 3.79 (0.40) | 46.3 (1.3) | 48.1 (2.0) | 44.8 (1.3) |
| Q4 | 58.5 (4.2) | 51.5 (5.0) | 65.1 (5.3) | 149.0 (5.8) | 133.4 (8.0) | 165.4 (8.3) | 89.8 (5.7) | 85.9 (6.0) | 92.6 (7.8) | 3.1 (0.3) | 2.64 (0.56) | 3.26 (0.36) | 47.6 (1.8) | 48.9 (2.4) | 46.5 (1.9) |
| P value for trend | 0.051 | 0.023 | 0.263 | 0.024 | 0.062 | 0.118 | 0.448 | 0.119 | 0.955 | 0.078 | 0.025 | 0.918 | 0.011 | 0.033 | 0.028 |

DPhP: diphenyl phosphate; BDCPP: bis(1,3-dichloro-2-propyl) phosphate; BCPP: bis-(1-chloro-2-propyl) phosphate; BCEP: bis-2-chloroethyl phosphate; DBUP: dibutyl phosphate
